# Artificial Clinic Intelligence (ACI): A Generative AI-Powered Modeling Platform to Optimize Patient Cohort Enrichment and Clinical Trial Optimization

**DOI:** 10.3390/cancers17213543

**Published:** 2025-11-01

**Authors:** Choong-Yong Ung, Cristina Correia, Zhuofei Zhang, Carter Caya, Shizhen Zhu, Daniel D. Billadeau, Hu Li

**Affiliations:** 1 Department of Molecular Pharmacology and Experimental Therapeutics, Mayo Clinic College of Medicine and Science, Rochester, MN 55905, USA; ung.choongyong@mayo.edu (C.-Y.U.); correia.cristina@mayo.edu (C.C.); zhan9971@umn.edu (Z.Z.); caya.carter@mayo.edu (C.C.); zhu.shizhen@mayo.edu (S.Z.); 2 Department of Bioinformatics and Computational Biology, University of Minnesota, Rochester, MN 55904, USA; 3 Department of Biochemistry and Molecular Biology, Mayo Clinic College of Medicine and Science, Rochester, MN 55905, USA; 4 Department of Immunology, Mayo Clinic College of Medicine and Science, Rochester, MN 55905, USA; billadeau.daniel@mayo.edu; 5 Division of Gastroenterology and Hepatology, Department of Medicine, Mayo Clinic, Rochester, MN 55905, USA; 6 Paul F. Glenn Center for Biology of Aging Research, Mayo Clinic, Rochester, MN 55905, USA

**Keywords:** clinical trial, clinical trial enrichment, artificial intelligence (AI), generative AI, modeling, patient stratification, patient enrichment, drug testing

## Abstract

**Simple Summary:**

Clinical trials are critical for assessing drug efficacy, toxicity, and potential long-term health effects in humans. Identifying in advance which patients are likely to benefit from a trial drug can enhance the success rate of clinical trials while reducing resource allocation and shortening the time for drug approval. In this perspective article we offer a roadmap on how to achieve Artificial Clinic Intelligence (ACI), a generative AI-driven virtual clinical trial enrichment framework which implements novel concepts for streamlining the selection of patients that will drive the most clinical benefits from a test drug. ACI generates synthetic patient data, models drug response across clinically diverse populations, and ranks the importance of clinical attributes underlying drug sensitivity. By evaluating the extent to which a prospective patient will benefit from a drug, ACI helps drive the success of clinical trials.

**Abstract:**

Clinical trial enrichment is the targeted recruitment of prospective individual patients with defined clinical characteristics who are likely to benefit from newly developed or repurposed drugs. This process is central to the success of clinical trials together with patient management and regulatory compliance. A main challenge in clinical trial enrichment lies in the recognition of a priori clinical parameters and information that informs drug efficacy or toxicity, particularly when intended for a broader unseen population. Although Artificial Intelligence (AI) approaches, especially large language models (LLMs), have been employed in many aspects of clinical trials, to our knowledge, there is no AI method that has been developed which offers a prospective prediction and assesses the extent to which a given therapeutic intervention benefits an unseen population. Here, we offer an outlook on how to build Artificial Clinic Intelligence (ACI), a generative AI (GAI)-powered modeling platform for modeling clinical trial enrichment. ACI generates synthetic patient data and models clinical trial enrichment to inform clinicians on key clinical parameters that are enriched in prospective patients prior to accrual.

## 1. Introduction

The goal of clinical trials is to ensure the effectiveness and safety of drugs to the target population. However, patient accrual and identification of suitable individuals that represent a target population is often time-consuming and costly and often fails to generalize to the larger unseen population. Thus, selecting the right patient population enriched with individuals who will benefit from a particular treatment is critical for the success of a clinical trial. This process is called clinical trial enrichment and is one of the key steps in clinical trial design [1]. Yet, most clinical trial designs focus mainly on a single or a few clinical variables such as genetic backgrounds to stratify patients, ignoring the complexity of and interactions between clinical variables defining a disease. Maximizing the effectiveness of prospective patient identification requires moving beyond a narrow set of clinical parameters (e.g., family history, genetics biomarkers, medical text records, etc.). Rather, to effectively identify clinical variables that can pinpoint prospective patients for a given therapeutic formulation requires the distillation of multimodal and multidimensional profiles (e.g., genomics and genome-wide gene expression profiles across disease types and drug responses). This process requires the use of sophisticated computational methods including state-of-the-art Artificial Intelligence (AI) approaches to identify latent patterns embedded within the experimental and clinical data, which cannot be accomplished using text-based descriptive data.

AI has been increasingly used in various clinical aspects in medicine from disease diagnosis [2] to drug matching to specific patients [3]. For example, large language models (LLMs) can enable clinicians to handle large volumes of medical data and enhance patient treatment management [4]. LLMs have also started to permeate clinical trial design and aid several tasks like patient matching, clinical trial planning, free text narrative embedding, writing tasks, as well as providing cognizant consent via chatbots [5]. While these AI-powered advancements are helpful, a key challenge in the design of clinical trials is to accurately identify prospective individuals while maximizing the likelihood of success of a clinical trial, which remains unresolved. Artificial Clinic Intelligence (ACI), our proposed AI-based solution, addresses this challenge by offering a generative AI-based ecosystem for clinical trial modeling. It estimates the benefit in a given therapeutic intervention in a selected population and prospectively predicts clinical parameters to enable trial enrichment of individuals most likely to respond.

In this perspective article we first identify challenges with the design of clinical trials, with a particular focus on clinical trial enrichment. However, aspects related to patient management and regulatory compliance are not the focus in this work. We outline existing AI tools that have been developed to support clinical trial design, examining their current limitations and identifying key unmet challenges that warrant further attention. Next, we introduce the components and novel concepts integral to the inner workings of ACI. We also describe the architecture of the ACI framework, how it operates, and identify foreseeable technical limitations. We use breast cancer (BC) as an illustrative example to demonstrate how ACI identifies clinical parameters that can inform clinicians a priori about which selected patient populations constitute prospective candidates before the recruitment process begins. Finally, we discuss the outlook of ACI and identify critical gaps that must be overcome to move forward.

## 2. How Clinical Trial Enrichment Influences Clinical Trial Success

Over the past few decades there has been a decline in the efficiency of drug discovery [6]. Drug discovery and development is a very costly enterprise (~USD 1.5–2.0 billion) and easily takes 10–15 years to bring a new drug to the market. Every investigational drug undergoes clinical trials before approval and entering the market, and candidates can fail at any stage. Drugs may fail at any phase of clinical trials with the largest cost to those that fail in the later phases of a clinical trial. Clinical trials are typically organized in consecutive Phases I-III and each phase has a particular goal. Phase I clinical trials usually recruit 20–100 healthy volunteers to assess drug safety and dosage to administer. Phase II trials recruit 100–300 volunteer patients and aim to confirm drug effectiveness and potential side effects. Phase III trials are often conducted at several medical centers with several hundreds to several thousand patients to evaluate effectiveness of newly developed drugs. Unlike Phases I and II, Phase III clinical trials employ a randomized controlled trial (RCT) design that randomly assign participants to groups that either receive genuine treatments or placebo treatments.

**Figure 1:**
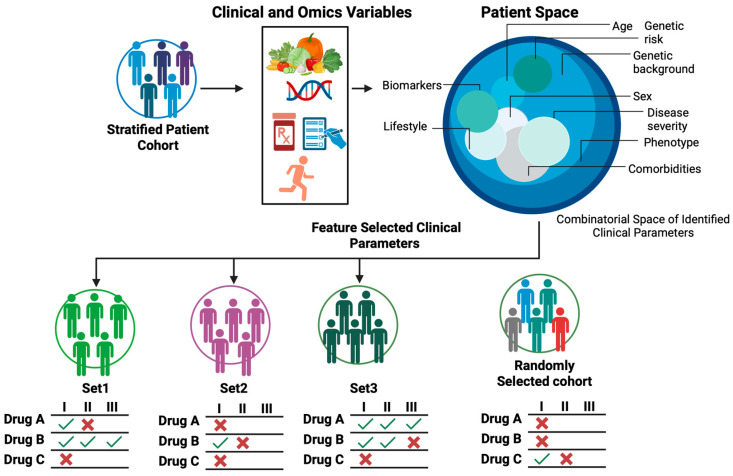
How clinical trial enrichment influences the success of clinical trials. Clinical trial enrichment is the process of recruiting prospective individuals who share similar clinical parameters and who are most likely to benefit from a trial drug. The figure illustrates how individuals grouped into a cohort influence the success of clinical trials (from Phases I to III) with drugs A, B, and C as trial drugs. In general, patients can be grouped into cohorts based on specific combinations of clinical parameters such as sex, race, ethnicity, family history, and lifestyle and omics profiles. Often a set of patients fail to respond to a therapeutic intervention, and the ability to distill the right combination of clinical parameters is key to determine clinical trial success. Only cohorts with the right combinations of clinical parameters enrich for likely responders, improving the probability of clinical trial success throughout clinical development. Figure generated with BioRender.

A key goal of clinical trial enrichment is to identify prospective individuals who are most likely to benefit from a particular trial drug, rather than blindly selecting any individual from a general population. However, patient selection and accrual are lengthy processes. While institutions such as Institutional Review Boards (IRB) serve a critical regulatory function in approving and initiating clinical trials [7], regulatory and administrative processes alone cannot provide further guidance on which clinical parameters should be considered to recruit prospective patients. Therefore, clinical trial enrichment is still a bottleneck that determines the success of clinical trials (Figure 1).

By aggregating patients who share similar clinical parameters into a trial cohort, researchers and clinicians can more clearly and quickly determine the extent to which a test drug benefits a group of patients, frequently with smaller studies. This strategy reduces inter-individual variability, boosts signal-to-noise, and increases the statistical power and the likelihood of detecting a real therapeutic effect. With the right patients and right clinical parameters, trials become shorter, cheaper, and more likely to succeed. The challenge is to define the right clinical parameters that enrich prospective patients for a given trial drug. In the next sections we offer our perspective on how to realize ACI, but before that we review some of the AI tools being used in clinical trials.

## 3. Current AI Approaches Used in Clinical Trial Design and Clinically Relevant Research

In silico clinical trials, also known as virtual trials, were proposed as early as the 2000s to advance drug development and improve clinical decision making [8,9,10]. In general, these virtual trials employ state-of-the-art computational approaches that create virtual patient populations and model drug safety and efficacy [11,12,13]. In addition, AI can add value by curating clinical data with generative language models [14], optimizing patient enrollment [15], matching patients to clinical trials [16], and improving patient management [17]. Nonetheless several substantial challenges remain unresolved, as mentioned in the previous section.

Overall, there are two main categories of AI used in clinical trials: deductive and generative. Deductive AI seeks to find patterns embedded in medical data while generative AI (GAI) has the power to generate synthetic clinical data with attributes conforming to the statistical distribution of the observed data. Deductive AI includes various forms of learning algorithms such as decision trees, support vector machines (SVMs), and deep neural networks (DNNs). On the other hand, GAI is a class of deep neural networks with specific architectures. For instance, a Generative Adversarial Network (GAN), which is one of the GAI approaches, consists of two AI systems known as the generator and discriminator. The generator creates synthetic data that resembles the real data, while the discriminator evaluates and determines whether the synthetic data conforms to the statistical distribution of the real data and is followed by providing feedback to the generator. We propose that rigorous generation and coupling of synthetic patient and drug response phenotypic data to emulate clinical trials is key to optimizing clinical trial design and increasing the likelihood of success. In the following sections, we focus our discussion on GAI-based applications in the context of clinic trials. Then we shift our attention to the role of GAI in synthetic data generation, and modeling of drug response profiles as proposed in the ACI.

### 3.1. Digital Twins: Virtual Replicas of Reality

With the advances of monitoring devices such as healthcare wearable and AI technologies, it is now possible to create digital twins, which are virtual replicas (or models) of any existing physical objects such as a human, system, or process using real-time data and simulation to mirror their properties and behaviors [18]. GAI has been applied to the generation of digital twins [19] and aids in identifying the optimal drug for a given patient [20]. Digital twins have been used to monitor the dynamic trajectories of various clinical variables [21], for example heart function [22], or physiological variables that enhance clinical decision making and support cancer patient care management in real-time [23].

### 3.2. Extract Knowledge from Medical Texts and Clinical Data via Large Language Models (LLMs)

LLMs are general-purpose language generators based on Generative Pre-trained Transformer (GPT) deep neural network technologies that have gained popularity recently to translate and generate human-like texts in various areas [24]. LLMs have the strength to extract and synthesize useful information from vast literature resources including large volumes of medica corpora. For instance, LLMs have been used to extract knowledge from clinical data, where clinical variables are described in text forms [25]. These text-based descriptive variables include patient information such as family history, lifestyle, disease states, sensitivity to a drug, and presence of specific genetic mutations alongside domain-specific knowledge pertaining to disease mechanisms and drug pharmacological characteristics. LLM tools such as PaLM [26], MedMCQA [27], PubMedQA [28], LiveQA [29], MedicationQA [30], and MultiMedQA [25] have been developed to retrieve and synthesize medical knowledge from vast medical texts to answer human queries via a chatbot. Other LLM tools such as ChatDoctor that facilitates patient–doctor interactions [31] and TrialGPT [32] that matches patients with the appropriate clinical trial have been developed.

### 3.3. Synthetic Patient Data—Generative AI to Model Clinical Outcomes

GAI not only has the power to generate text data but in principle it can be applied to generate data of any modality. GAI has been broadly used to produce synthetic medical imaging data [33,34,35]. More recently GAI has also been used to generate synthetic gene expression data [36]. Eckardt et al. recently employed CTAB-GAN to generate synthetic acute myeloid leukemia patient cohorts using cytogenetic and sequencing data to model patient survival [37].

### 3.4. Synthetic Animal Data—Generative AI to Model Responses to Chemicals

Chen et al. recently developed Tox-GAN, a GAN-based tool to derive novel results underlying toxics exposure using existing animal treatments without the need to perform additional toxicological experiments [38]. Tox-GAN was trained with molecular descriptors that describe the chemical structure of 170 compounds across multiple dosages of exposure, and transcriptomics data collected after exposure to these toxicants. The goal of Tox-GAN is to generate synthetic transcriptomics data that recapitulate gene expression profiles induced by toxicants. The trained Tox-GAN model permits transcriptomic profile inference based on chemical information of toxicants. In their subsequent study, and using a similar computational strategy, Chen et al. developed AnimalGAN to generate synthetic animal data to assess hepatotoxicity of drugs [39].

## 4. Gaps of Current AI Approaches in Clinical Trials

Though useful in forecasting disease progression [40] and promoting individualized treatments [41], digital twins only capture one physical patient at a time, and thus fail to generalize to patients who are not yet subjected to a treatment. Essentially, digital twins cannot replicate a twin if real data on real physical objects is missing. Although digital twins are maturing as individual-level simulators in healthcare advance and offer valuable support for precision decision making, digital twins alone are not sufficient to solve the challenge of cohort selection in clinical trials [21].

While LLMs are valuable for enhancing doctor-patient interactions and extraction of medical knowledge, their utility is largely confined to handling text-based medical data and have limited capability to extract biological information hidden within numerical data especially when it comes to phenotypic profiles that are captured via high-throughput tabulated data.

Notwithstanding that GAI tools such as Tox-GAN [38] and AnimalGAN [39] have great promise in assessing drug safety and efficacy, they lack the capability to inform which clinical parameters are useful for patient stratification or the criteria needed to define and monitor the efficacy of a trial drug. New concepts and AI-based integration are therefore required to address the pressing challenges in clinical trial design.

## 5. Artificial Clinic Intelligence (ACI): A Working Draft to Address Challenges in Clinical Trial Enrichment

To address the aforementioned challenges in clinical trial enrichment and limitations of current AI tools used in clinical trials, we propose a solution called Artificial Clinic Intelligence (ACI), as a working draft for building a generative AI-based modeling ecosystem that allows researchers and clinicians to prospectively identify clinical parameters to enrich prospective patients who are likely to benefit from a given test drug in a clinical trial.

**Figure 2:**
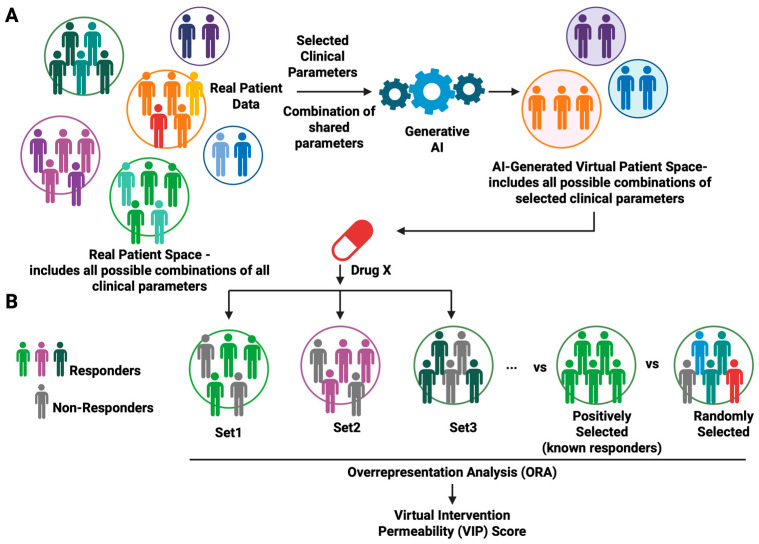
Core concepts of Artificial Clinic Intelligence (ACI). (**A**) Patient Virtual Space (PVS). Real patient space consists of subpopulations made of individuals of all possible combinations of all clinical parameters. Such space is astronomically huge and costly to explore. In contrast, PVS is a simplified AI-generated model of patient space consisting of subpopulations made of virtual patients of all possible combinations of a confined set of clinical parameters. As it is not possible to have clinical data for all real human individuals, PVS is built by generative AI using available real patient data. PVS is thus an exploratory model composed of virtual patients for clinical investigation. (**B**) Virtual Intervention Permeability (VIP). VIP quantifies the extent to which a virtual cohort, defined by individual patients grouped with similar clinical parameters, exhibit the desired outcomes to a trial drug (Drug X). Using scoring schemes based on over-representation analysis (ORA), we can estimate the enrichment of a given virtual cohort against a ground-truth cohort (all virtual responders regardless of their clinical parameters) relative to a randomly selected cohort (randomly selected virtual patients). The resulting VIP scores serve as a clinical trial enrichment metric where high VIP scores indicate cohorts enrichened in relevant clinical parameters that can guide recruitment of prospective patients most likely to benefit from a trial drug. Figure generated with BioRender.

Overall, ACI operates based on two novel concepts: Patient Virtual Space (PVS) and Virtual Intervention Permeability (VIP) (Figure 2).

### 5.1. Patient Virtual Space (PVS)

Patient heterogeneity is a key bottleneck in clinical trials. In principle, each patient should be described with a complete set of clinical parameters that include but is not limited to family history, genetics, lifestyle, physiological monitoring, and other omics measures. However, achieving an in-depth description is costly. Conventionally, patients are stratified based on either a single clinical variable or combination of a set of multiple clinical variables. Finding the right combinations of clinical parameters to better stratify patients during the recruitment phase in clinical trials remains challenging. To address this problem, we put forward a novel concept called Patient Virtual Space—PVS (Figure 2A). Real patient space is complex and high-dimensional, defined not only by the sample size but by the full set of all possible clinical variable combinations in a population. This parallels chemical space which spans all chemical compounds, natural or synthetic, often used in chemical screening. Conversely, the Patient Virtual Space is a searchable n-dimensional space that spans a confined set of clinical variables defining an individual to encompass its heterogeneity: age, sex, disease severity, biomarker, ethnic group, geographical regions, nation, genetic history, genetic variation, genetic risk, disease stage, comorbidities, etc. In contrast to a real patient population, PVS is an exploratory virtual model of the patient population.

Framed in ML terms, PVS is a feature selection over patient covariates. Rather than using all features, we can build simpler yet reasonably well-performing models with a smaller set of features. Although PVS is simpler than the real patient space, it is by no means small, as the number of possible combinations of selected clinical parameters is still huge. Therefore, to identify the right combinations of clinical parameters that enrich prospective patients for a trial drug, an iterative parameter enrichment process is required.

### 5.2. Virtual Intervention Permeability

Two questions need to be addressed before a trial drug passes clinical trial and enters the market: (i) To what extent does a drug exhibit effectiveness in the overall population? (ii) Which subpopulation (ethnic groups, age, sex, genetic background, disease stage, etc.) benefits most from a specific drug treatment? The concept of Virtual Intervention Permeability (VIP) is designed to address these questions. We use the diffusion analogy of a drop of dye diffusing though a piece of paper to help “visualize” this concept. Imagine a drop of dye falling onto the center of a paper made of different types of fibers with different permeability to the dye. Over time, the dye will diffuse to areas made of fibers with high permeability while areas made of fibers with poor permeability resist the diffusion of the dye. Here, the paper represents general human population while different fibers that make up the paper represent different human subpopulations for individuals who share a similar clinical parameter set. The dye represents a trial drug and permeability of the dye represents the efficacy of a drug to a given subpopulation.

### 5.3. VIP Scoring Schemes to Score Virtual Patient Subpopulations Enriched for Likely Responders

VIP enables the modeling of virtual patient subpopulations and associated clinical parameters. To achieve this goal, a scoring scheme akin to over-representation analysis (ORA) in pathway enrichment [42] can be designed to score subpopulations enriched with prospective virtual patients. (Figure 2B). Methods used in ORA such as the binomial test, hypergeometric test, and Fisher’s exact test can be employed. Because an individual virtual patient can potentially be grouped into different subpopulations due to overlapping of some of the clinical characteristics, just like one gene can be assigned to multiple biological pathways, the goal of ORA is to find which virtual subpopulations are statistically enriched with individuals who show the desirable drug response (e.g., disappearance of disease markers and resolution of symptoms). ORA will be conducted on the set of virtual individuals that show the desired responses to a trial drug (positive cohort), together with a negative reference compromising random virtual patients to assess statistical significance. Virtual patient subpopulations with high VIP scores, i.e., statistically enriched, will nominate the clinical parameters essential for stratifying prospective beneficiaries.

## 6. The Architecture and the Working of ACI Framework

Unlike current AI and/or statistical predictive models that are built with existing patient cohorts and perform a prediction when encountering a new patient, ACI uses the concept of VIP to realize a priori prediction and assesses to what extent a given therapeutic intervention benefits a stratified population while also identifying which stratification criteria leads to the highest intervention benefit in unseen patients.

While current AI approaches rely heavily on LLMs to mine medical texts like electronic health records (EHRs) for recruitment criteria, ACI provides prior enrichment—real clinical parameters—to researchers and clinicians, facilitating more effective patient recruitment and higher trial success. In other words, ACI uses GAI to provide a *prospective* modeling strategy based on AI-generated drug and genetic activities rather than medical text to prioritize clinical parameters that aid patient stratification. We reason that this is a more realistic approach to capture patient heterogeneity and clinical nuances in unseen populations. Next, we describe the overall architecture of ACI and its feasibility.

**Figure 3:**
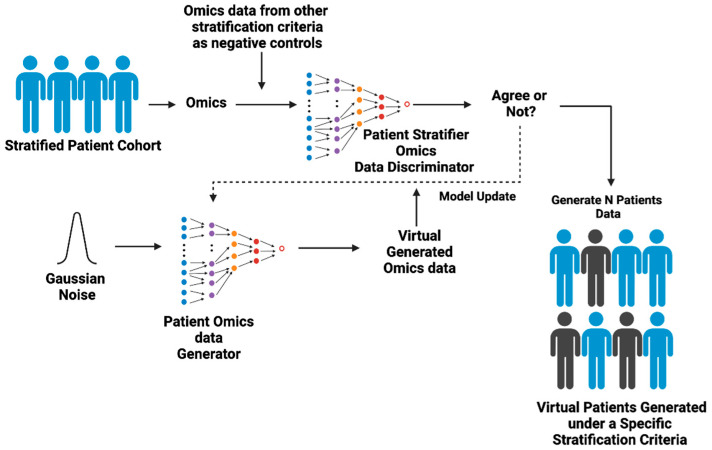
The workings of Virtual Patient GAI. Virtual Patient GAI (VP-GAI) models trained with real clinical data for patients with demographic and multi-omics data available. First, the discriminator component learns the statistical distribution of data attributes that help discriminate a disease (e.g., breast cancer) and its subtypes and uses the classification approach to assess the performance of the discriminator. On the other hand, the generator portion learns to generate synthetic patient data starting from random noise. In the beginning, the generator produces low-quality data that the discriminator can easily identify as fake. However, over many iterations of discriminator–generator interactions, the generator gradually learns to produce synthetic patient data that the discriminator no longer reliably distinguishes as “fake”. Once trained, the VP-GAI can easily generate thousands of individual synthetic patient data that conform to the Patient Virtual Space of a disease. Figure generated with BioRender.

**Figure 4:**
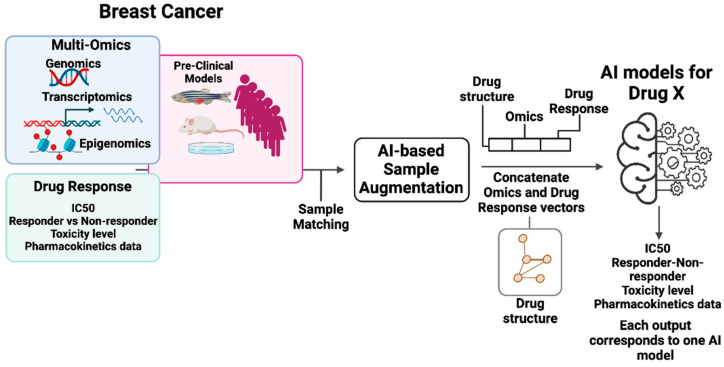
Drug AI module for ACI. This module includes numerous Drug AI models, with one drug per each model. Here, drug X is used as an illustrative case. The inputs include multi-omics, drug response outputs, and data from pre-clinical models. In the training phase concatenated feature vectors including information on drug X structure, matched drug response profiles, and multi-omics data for an individual patient, cell line, or animal model are used. The training dataset can be augmented using generative AI approaches. A Drug AI model will use a classification or regression approach depending on data availability, and performance metrics are used to assess model performance. Once trained, the Drug AI model will predict a response phenotype from a drug structure and an individual’s omics data. Figure generated with BioRender.

ACI is composed of two interactive GAI components: Virtual Patient GAI (VP-GAI) (Figure 3) and Drug AI (Figure 4). Integrative modeling of these two GAI components solidifies ACI as an AI-based modeling system that enables the identification of patient populations that will benefit from a particular treatment and simultaneously extracts the clinical information contributing to its success.

### 6.1. Virtual Patient GAI (VP-GAI)

The goal of this component is to generate synthetic patient data to build a Patient Virtual Space (PVS) for modeling clinical parameters predictive of benefit from the trial drug. VP-GAI uses publicly available clinical and omics data as inputs for training (Figure 3). For the sake of simplicity, we consider transcriptomics data with well annotated clinical information (e.g., age, ethnic group, disease and medical history, genetic background, etc.) as input data. For example, for a trial drug to be tested on its therapeutic effects on breast cancer (BC), available transcriptomics data from BC patient cohorts will be used to train a VP-GAI model. On the other hand, transcriptomics data from other diseases will be used as negative training sets. Here, the discriminator of VP-GAI will learn the statistical distribution of gene expression activities of BC from other diseases. The performance of the discriminator can be evaluated via classification. On the other hand, the generator of VP-GAI aims to generate synthetic BC transcriptomics data that recapitulates the statistical distribution of clinical attributes in the training dataset. Gaussian noise (random noise) can be added to the input data and subjected to the discriminator to assess whether the generated data captures the statistical behavior of BC patients. At the beginning, the synthetic data generated by the generator is very much dissimilar to real BC patient data. However, through iterative learning and improving discrimination–generation procedure, the generator eventually generates synthetic data that conforms to the statistical distribution of attributes specific to BC patients. Once the generator has achieved the desired generative performance, various synthetic BC patient data can be generated to build a patient space for BC. At this stage, we have a generic VP-GAI with respect to a disease (breast cancer in this case). Starting with this generic VP-GAI model, using transfer learning with a similar discrimination–generation training process, the next phase is to build VP-GAI subpopulation models with respect to specific combinations of clinical parameters.

### 6.2. Drug AI Model

The goal of this component is to develop a GAI model that recapitulates response phenotypes with respect to a given drug (Figure 4). Multi-omics data (e.g., genomics, transcriptomics, proteomics, and epigenomics) of individual patients with matched response phenotype data correspond to a given drug, i.e., IC50 measured as a continuous variable or binned as “responder vs. non-responder” can be used as inputs to train a Drug AI model. Resources such as DrugBank [43], PubChem [44], Gene Expression Omnibus (GEO) [45], Cancer Cell Line Encyclopedia (CCLE) [46] and DepMap [46,47], Genomics of Drug Sensitivity in Cancer (GDSC) [48], and LINCS L1000 [49] can be used to enrich the input dataset. Whenever needed, a GAI approach like VP-GAI described above can be employed to augment the dataset for training. To increase the specificity of data that captures pharmacological behaviors of a drug, the input vector can be constructed by concatenating with molecular descriptors that describe the structure of the drug (e.g., SMILES, MOL, and SDF) together with omics data and response phenotype. Learning performance can be evaluated via classification or regression strategies. The resulting trained Drug AI model is generic, meaning given the chemical structure of a drug and omics data, the model outputs a drug-responsive phenotype. In other words, the goal of the Drug AI model is to predict response phenotypes (sensitive or resistant) or the extent of response (e.g., IC50) given a drug structure and omics data of an individual. Alternatively, one can also build drug class-specific AI models such as GPCR antagonists, which are expected to deliver greater specificity and performance than generic models.

### 6.3. How ACI Works: Breast Cancer (BC) as an Illustrative Example

We use breast cancer (BC) as an illustrative example to elaborate how ACI works; however, ACI is disease agnostic and applicable to rare diseases or other biological contexts. Let us start with a general BC patient cohort with clinical data containing the following attributes: age, sex, ethnic group, disease stage, estrogen receptor (ER) status, treatment history, survival outcomes, and omics data (in particular genomics and transcriptomics data). This BC cohort can then be further stratified into sub-cohorts based on the available clinical parameters such as age, disease state, and ER status (e.g., presence or absence of ER or certain types of mutations on ER gene). Starting with generic BC VP-GAI model described in the section above, omics data with respect to each sub-cohort will be used as input data to train cohort-dependent VP-GAI with the goal to generate synthetic patient data that constitute the Patient Virtual Space that represents each sub-cohort. The training and testing process can be conducted as described above.

As for the Drug AI component, suppose drug X is the drug to be evaluated in a clinical trial. There are two key questions to be addressed: (i) Will drug X be effective in most if not all sub-cohorts? (ii) Will a specific patient subpopulation benefit more from drug X and thus demonstrate a high Virtual Intervention Permeability (VIP) score? Or vice versa? To address these questions, synthetic patient data generated from each cohort-dependent VP-GAI will be subjected to the Drug X AI model and the VIP score will be computed using over-representation analysis (ORA) approaches described above with respect to individual patients in each subpopulation. In this way, we can identify high VIP score sub-cohorts enriched with effective individuals. Because clinical parameter sets with respect to high VIP score subpopulations are known, this means cohorts for patients recruited based on these clinical parameters are most likely prospective individuals for drug X. The process of patient stratification described above can be repeated using other clinical parameters, such as presence of specific mutations or expression activities of specific genes that had been demonstrated to drive BC. Again, each cohort or sub-cohort will be subjected to VP-GAI and each of these virtual cohorts will be tested on the efficacy of drug X using the Drug AI modeling strategy. Based on the VIP scores, the drug modeling process will allow us to identify subpopulations with the right combinations of clinical parameters enriched with prospective individuals that will benefit the most from drug X.

**Figure 5:**
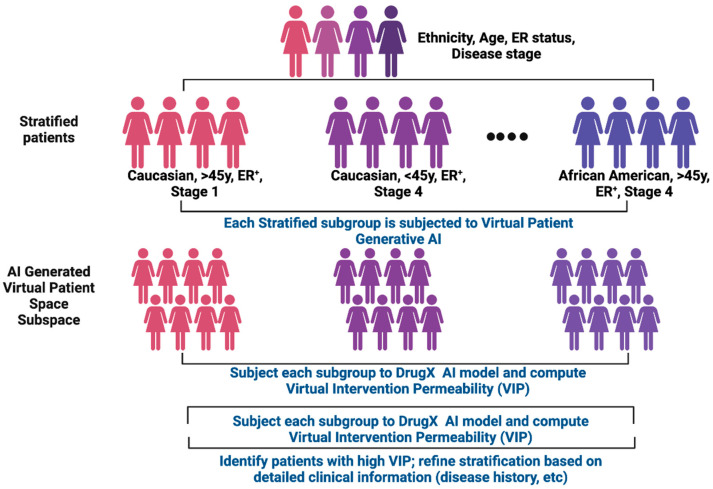
Clinical parameter distillation within ACI. The “distillation” process begins with virtual cohorts showing high VIP scores for drug X, where drug X is being tested in a clinical trial. These cohorts are iteratively stratified by combinatorial subsets of clinical parameters with VIP scoring recomputed at each step. Parameters contributing the least to VIP scoring will be eliminated whereas informative parameters are retained for subsequent iterations. The final distilled set represents a consensus of clinical parameters to guide the recruitment of prospective trial participants. Figure generated with BioRender.

The next step is to “distill” the combinations of clinical parameters that best enrich prospective individuals (e.g., age, sex, specific mutations on a particular gene, expression levels of specific genes, etc.). We called this process clinical parameter distillation (Figure 5). The “distillation” process will be performed iteratively, and in each iteration, certain clinical parameters will be eliminated while others retained for next iteration step. This process is akin to feature selection to determine features that contribute to the performance of a machine learning model. The final distilled clinical parameters are a set of consensus parameters that can be used as guidelines for patient selection in the process of clinical trial enrichment. ACI modeling therefore provides a priori information to inform clinicians as to which clinical parameters should be considered in the design of clinical trials on which prospective patients should be recruited and who should be excluded (i.e., patients who are predicted not to benefit from a test drug). This not only helps save costs and time but more importantly, for the first time, offers a priori guidance to address the challenges of clinical trial enrichment for a better and more effective clinical trial design.

### 6.4. Feasibility and Technical Limitations of ACI

As described above, the core engine for both VP-GAI and Drug AI is based on generative AI (GAI). As also discussed above, GAI, especially Generative Adversarial Network (GAN), has been used to build generative models to emulate chemical toxicity (e.g., Tox-GAN [38] and AnimalGAN [39]). Nair et al. used GAN to model biomarker profiles for under-represented race/ethnicity groups [50]. Their GAN model identified joint distributions of a panel of 16 diabetes-relevant biomarkers from the National Health and Nutrition Examination Survey, which contains laboratory and clinical biomarker data from a population-based sample of individuals of all ages, racial groups, and ethnicities. Their work showed it is feasible to use GAN to generate virtual patient data, in addition to AnimalGAN for the feasibility to generate virtual animal data.

Given that ACI uses similar GAN architecture and training procedures, the approach is in principle highly feasible. We therefore anticipate that ACI will encourage researchers to adopt this strategy for clinical trial modeling in the near term.

Compared with other existing methods, ACI incorporates innovative concepts like Patient Virtual Space (PVS), Virtual Intervention Permeability (VIP), and clinical parameter distillation. Unlike other GAN tools that generate synthetic data (animal or patient virtual data) for one or a few clinical parameters, PVS covers all possible combinations of a defined number of clinical parameters. On the other hand, VIP informs which virtual subpopulations are enriched with prospective individuals, and through clinical variable distillation informs clinical parameters that should be used to enrich prospective individuals who would benefit from a test drug.

As with most other GAI including GAN models, we identified several foreseeable technical limitations in ACI. For instance, ACI training can be unstable with risk of overfitting or model collapse, especially with small data size and high-dimensional inputs. Wasserstein GAN (WGAN) and Lipschitz-style regularization as implemented in AnimalGAN [39] can be used to stabilize GANs and mitigate collapse of gradients in the models. Also, the models can be sensitive to the choice of hyperparameters such as learning rate and batch size. This needs to be explored during model building stage.

## 7. ACI Checks and Regulatory Checkpoints

GAI has tremendous potential to aid in the creation of outputs but assessing their validity is key to drive clinician trust. ACI is designed to detect unreliable predictions. To that end it uses generator–discriminator strategy to mitigate hallucinations, a situation when a generative AI model produces incorrect and misleading outputs. Another mitigating strategy is to incorporate parallel AI systems trained in different datasets to detect anomalies and ensure the validity of outputs. Recently, Olsson et al. [51] implemented a strategy to estimate uncertainty in AI models. Using conformal prediction [52], a mathematical framework to guarantee an error rate is bound to a specific pre-defined level, it is possible to set up confidence levels and include quality control steps in AI methods. Implementing such check ingredients in ACI will ensure its outputs are aligned with clinical realities.

AI readiness is key for clinical implementation of ACI and hinges on two pillars: data operability and regulatory compliance. Data interoperability can be accomplished by sharing standards across organizations in healthcare. Here, GAI can play a pivotal role in building generative common data elements (GenCDEs) that are reusable across institutions and support federated learning. Assessing interoperability is also highly desirable. This can be accomplished in ACI by using a scoring system and performance metrics. For example, Long et al. [53] described an approach based on data matching, completeness, and compliance.

Like other GAI tools implemented in clinic, ACI encounters similar regulatory concerns. Although using GAIs such as GANs to create synthetic patient data to model drug-response can help promote clinical findings, it also raises regulatory questions related with privacy, scientific validity, data integrity, and AI governance. As GAI models learn the distribution of real patient data, there is a risk that the generators will leak unique or rare patterns. Another concern of using synthetic data is bias amplification as well as model degradation. However, it is also essential to emphasize that GAI-generated patient data are for simulation and method development only and not a replacement and cannot serve as confirmatory evidence. The U.S. Department of Health and Human Services has outlined six principles of trustworthy AI focused on patient safety, human rights obligations, and academic integrity defining the FAVES framework—Fair, Appropriate, Valid, Effective, and Safe [54,55]. Developers are encouraged to deploy safeguards and disclose model architecture, training and testing datasets, performance, and source codes when feasible. For further reading we refer to FDA’s AI draft guidance [56].

## 8. Discussion and Conclusions

We discussed challenges in the design of the process of clinical trial enrichment for recruiting prospective patients who are likely to benefit from a test drug. We also described examples of current AI tools used in clinical trials, discussed the missing links, and our motivation to propose the ACI framework. Although we used breast cancer as an illustrative example, ACI strategy is agnostic to both disease type and therapeutic agent, enabling broad applicability including application to rare diseases such as cystic fibrosis and sickle cell disease. The power of ACI is that it can generate synthetic patient data that represents the virtual space of a cohort stratified based on various combinations of clinical attributes. Moreover, ACI also evaluates the extent of individual synthetic patients who will benefit from the treatment of a given drug, an evaluation metric we called Virtual Intervention Permeability (VIP). By ranking cohorts (or sub-cohorts) with high or low VIP scores, we will be able to identify the right combinations of clinical attributes that define prospective patients. This means ACI can provide a priori information to clinicians to better design who should be recruited to maximize clinical trial enrichment processes. All these imply that ACI can be tailored at different phases of clinical trials, especially Phases II and III where recruitment of right individual patients is needed to confirm drug effectiveness and toxicity, in contrast to Phase I in which the focus is on drug safety and dosage applied to the general population.

However, the practicality of ACI heavily relies on the completeness of the data encompassing various clinical aspects of individual patients, including multimodal omics data as well as clinical follow-up, both of which require informed consent. Nonetheless, increasing awareness of the value of multimodal clinical data, and their potential to enhance the success of future clinical trials, will encourage the medical community to prioritize the generation, annotation, and collection of such data. This does not imply that we must endlessly generate matched clinical data for every patient. In fact, synthetic patient data generated by a well-trained ACI system is clinically valuable and can be generated in large amounts at low cost to emulate clinical trials. This implies that when combined with real patient data, ACI-generated synthetic patient data can serve as an additional resource to complement medical research beyond clinical trial modeling.

In summary, our proposed ACI system supports clinicians in identifying suitable prospective patients, thereby enhancing clinical trial success rates, streamlining clinical trial operations, and overall reducing cost burdens. ACI also offers a flexible AI-based ecosystem, with Virtual Patient GAI and Drug AI components functioning as standalone models. This plug-and-play architecture allows researchers and clinicians to integrate available medical knowledge into the ACI system, facilitating targeted solutions to specific medical problems.

## Data Availability

No new data were created or analyzed in this study. Data sharing is not applicable to this article.

## Abbreviations

The following abbreviations are used in this manuscript:

ACI	Artificial Clinic Intelligence
AI	Artificial Intelligence
LLM	Large language model
GAI	Generative AI
RCT	Randomized controlled trial
IRB	Institutional Review Boards
SVM	Support vector machine
DNN	Deep neural networks
GPT	Generative pre-trained Transformer
PVS	Patient Virtual Space
EHR	Electronic health records
BC	Breast cancer
ER	Estrogen receptor
VIP	Virtual Intervention Permeability

## References

[1] Thall P.F. (2021). Adaptive Enrichment Designs in Clinical Trials. Annu. Rev. Stat. Appl..

[2] D’Adderio L., Bates D.W. (2025). Transforming diagnosis through artificial intelligence. NPJ Digit. Med..

[3] Johnson K.B., Wei W.Q., Weeraratne D., Frisse M.E., Misulis K., Rhee K., Zhao J., Snowdon J.L. (2021). Precision Medicine, AI, and the Future of Personalized Health Care. Clin. Transl. Sci..

[4] Vrdoljak J., Boban Z., Vilovic M., Kumric M., Bozic J. (2025). A Review of Large Language Models in Medical Education, Clinical Decision Support, and Healthcare Administration. Healthcare.

[5] Ghim J.L., Ahn S. (2023). Transforming clinical trials: The emerging roles of large language models. Transl. Clin. Pharmacol..

[6] Scannell J.W., Blanckley A., Boldon H., Warrington B. (2012). Diagnosing the decline in pharmaceutical R&D efficiency. Nat. Rev. Drug Discov..

[7] Martin S.L., Allman P.H., Dugoff L., Sibai B., Lynch S., Ferrara J., Aagaard K., Zornes C., Wilson J.L., Gibson M. (2023). Outcomes of shared institutional review board compared with multiple individual site institutional review board models in a multisite clinical trial. Am. J. Obstet. Gynecol. MFM.

[8] Holford N.H., Kimko H.C., Monteleone J.P., Peck C.C. (2000). Simulation of clinical trials. Annu. Rev. Pharmacol. Toxicol..

[9] An G. (2004). In silico experiments of existing and hypothetical cytokine-directed clinical trials using agent-based modeling. Crit. Care Med..

[10] Clermont G., Bartels J., Kumar R., Constantine G., Vodovotz Y., Chow C. (2004). In silico design of clinical trials: A method coming of age. Crit. Care Med..

[11] Pappalardo F., Russo G., Tshinanu F.M., Viceconti M. (2019). In silico clinical trials: Concepts and early adoptions. Brief. Bioinform..

[12] Chopra H., Shin D.K., Munjal K., Dhama K., Emran T.B. (2023). Revolutionizing clinical trials: The role of AI in accelerating medical breakthroughs. Int. J. Surg..

[13] Harrer S., Shah P., Antony B., Hu J. (2019). Artificial Intelligence for Clinical Trial Design. Trends Pharmacol. Sci..

[14] Xu R., Cui H., Yu Y., Kan X., Shi W., Zhuang Y., Jin W., Ho J., Yang C. (2023). Knowledge-infused prompting: Assessing and advancing clinical text data generation with large language models. arXiv.

[15] Chow R., Midroni J., Kaur J., Boldt G., Liu G., Eng L., Liu F.F., Haibe-Kains B., Lock M., Raman S. (2023). Use of artificial intelligence for cancer clinical trial enrollment: A systematic review and meta-analysis. J. Natl. Cancer Inst..

[16] Nievas M., Basu A., Wang Y., Singh H. (2024). Distilling large language models for matching patients to clinical trials. J. Am. Med. Inform. Assoc..

[17] Bajwa J., Munir U., Nori A., Williams B. (2021). Artificial intelligence in healthcare: Transforming the practice of medicine. Future Healthc. J..

[18] Boschert S., Rosen R. (2016). Digital twin—The simulation aspect. Mechatronic Futures: Challenges and Solutions for Mechatronic Systems and Their Designers.

[19] Grieves M.W. (2019). Virtually intelligent product systems: Digital and physical twins. Complex Systems Engineering: Theory and Practice.

[20] Bordukova M., Makarov N., Rodriguez-Esteban R., Schmich F., Menden M.P. (2024). Generative artificial intelligence empowers digital twins in drug discovery and clinical trials. Expert. Opin. Drug Discov..

[21] Venkatesh K.P., Brito G., Kamel Boulos M.N. (2024). Health Digital Twins in Life Science and Health Care Innovation. Annu. Rev. Pharmacol. Toxicol..

[22] Baillargeon B., Rebelo N., Fox D.D., Taylor R.L., Kuhl E. (2014). The Living Heart Project: A robust and integrative simulator for human heart function. Eur. J. Mech. A Solids.

[23] Kaul R., Ossai C., Forkan A.R.M., Jayaraman P.P., Zelcer J., Vaughan S., Wickramasinghe N. (2023). The role of AI for developing digital twins in healthcare: The case of cancer care. Wiley Interdisciplinary Reviews: Data Mining and Knowledge Discovery.

[24] Brown T., Mann B., Ryder N., Subbiah M., Kaplan J.D., Dhariwal P., Neelakantan A., Shyam P., Sastry G., Askell A. (2020). Language models are few-shot learners. Adv. Neural Inf. Process. Syst..

[25] Singhal K., Azizi S., Tu T., Mahdavi S.S., Wei J., Chung H.W., Scales N., Tanwani A., Cole-Lewis H., Pfohl S. (2023). Large language models encode clinical knowledge. Nature.

[26] https://arxiv.org/abs/2204.02311.

[27] Pal A., Umapathi L.K., Sankarasubbu M. Medmcqa: A large-scale multi-subject multi-choice dataset for medical domain question answering. Proceedings of the ACM Conference on Health, Inference, and Learning.

[28] Jin Q., Dhingra B., Liu Z., Cohen W.W., Lu X. (2019). Pubmedqa: A dataset for biomedical research question answering. arXiv.

[29] Abacha A.B., Agichtein E., Pinter Y., Demner-Fushman D. Overview of the medical question answering task a TREC 2017 LiveQA. Proceedings of the Text REtrieval Conference (TREC) 2017.

[30] Abacha A.B., Mrabet Y., Sharp M., Goodwin T.R., Shooshan S.E., Demner-Fushman D. (2019). Bridging the gap between consumers’ medication questions and trusted answers. MEDINFO 2019: Health and Wellbeing e-Networks for All.

[31] Li Y., Li Z., Zhang K., Dan R., Jiang S., Zhang Y. (2023). ChatDoctor: A Medical Chat Model Fine-Tuned on a Large Language Model Meta-AI (LLaMA) Using Medical Domain Knowledge. Cureus.

[32] Jin Q., Wang Z., Floudas C.S., Chen F., Gong C., Bracken-Clarke D., Xue E., Yang Y., Sun J., Lu Z. (2024). Matching Patients to Clinical Trials with Large Language Models. arXiv.

[33] Gong C., Jing C., Chen X., Pun C.M., Huang G., Saha A., Nieuwoudt M., Li H.X., Hu Y., Wang S. (2023). Generative AI for brain image computing and brain network computing: A review. Front. Neurosci..

[34] Arora A., Arora A. (2022). Generative adversarial networks and synthetic patient data: Current challenges and future perspectives. Future Healthc. J..

[35] Lang O., Yaya-Stupp D., Traynis I., Cole-Lewis H., Bennett C.R., Lyles C.R., Lau C., Irani M., Semturs C., Webster D.R. (2024). Using generative AI to investigate medical imagery models and datasets. EBioMedicine.

[36] Lee M. (2023). Recent advances in generative adversarial networks for gene expression data: A comprehensive review. Mathematics.

[37] Eckardt J.N., Hahn W., Rollig C., Stasik S., Platzbecker U., Muller-Tidow C., Serve H., Baldus C.D., Schliemann C., Schafer-Eckart K. (2024). Mimicking clinical trials with synthetic acute myeloid leukemia patients using generative artificial intelligence. NPJ Digit. Med..

[38] Chen X., Roberts R., Tong W., Liu Z. (2022). Tox-GAN: An Artificial Intelligence Approach Alternative to Animal Studies-A Case Study With Toxicogenomics. Toxicol. Sci..

[39] Chen X., Roberts R., Liu Z., Tong W. (2023). A generative adversarial network model alternative to animal studies for clinical pathology assessment. Nat. Commun..

[40] Allen A., Siefkas A., Pellegrini E., Burdick H., Barnes G., Calvert J., Mao Q., Das R. (2021). A digital twins machine learning model for forecasting disease progression in stroke patients. Appl. Sci..

[41] Kamel Boulos M.N., Zhang P. (2021). Digital Twins: From Personalised Medicine to Precision Public Health. J. Pers. Med..

[42] de Leeuw C.A., Neale B.M., Heskes T., Posthuma D. (2016). The statistical properties of gene-set analysis. Nat. Rev. Genet..

[43] Knox C., Wilson M., Klinger C.M., Franklin M., Oler E., Wilson A., Pon A., Cox J., Chin N.E.L., Strawbridge S.A. (2024). DrugBank 6.0: The DrugBank Knowledgebase for 2024. Nucleic Acids Res..

[44] Kim S., Chen J., Cheng T., Gindulyte A., He J., He S., Li Q., Shoemaker B.A., Thiessen P.A., Yu B. (2023). PubChem 2023 update. Nucleic Acids Res..

[45] Clough E., Barrett T. (2016). The Gene Expression Omnibus Database. Methods Mol. Biol..

[46] Barretina J., Caponigro G., Stransky N., Venkatesan K., Margolin A.A., Kim S., Wilson C.J., Lehar J., Kryukov G.V., Sonkin D. (2012). The Cancer Cell Line Encyclopedia enables predictive modelling of anticancer drug sensitivity. Nature.

[47] Ghandi M., Huang F.W., Jane-Valbuena J., Kryukov G.V., Lo C.C., McDonald E.R., Barretina J., Gelfand E.T., Bielski C.M., Li H. (2019). Next-generation characterization of the Cancer Cell Line Encyclopedia. Nature.

[48] Yang W., Soares J., Greninger P., Edelman E.J., Lightfoot H., Forbes S., Bindal N., Beare D., Smith J.A., Thompson I.R. (2013). Genomics of Drug Sensitivity in Cancer (GDSC): A resource for therapeutic biomarker discovery in cancer cells. Nucleic Acids Res..

[49] Subramanian A., Narayan R., Corsello S.M., Peck D.D., Natoli T.E., Lu X., Gould J., Davis J.F., Tubelli A.A., Asiedu J.K. (2017). A Next Generation Connectivity Map: L1000 Platform and the First 1,000,000 Profiles. Cell.

[50] Nair R., Mohan D.D., Frank S., Setlur S., Govindaraju V., Ramanathan M. (2023). Generative adversarial networks for modelling clinical biomarker profiles with race/ethnicity. Br. J. Clin. Pharmacol..

[51] Olsson H., Kartasalo K., Mulliqi N., Capuccini M., Ruusuvuori P., Samaratunga H., Delahunt B., Lindskog C., Janssen E.A.M., Blilie A. (2022). Estimating diagnostic uncertainty in artificial intelligence assisted pathology using conformal prediction. Nat. Commun..

[52] Vovk V., Gammerman A., Shafer G. (2005). Algorithmic Learning in a Random World.

[53] Long R.A., Ballard S., Shah S., Bianchi O., Jones L., Koretsky M.J., Kuznetsov N., Marsan E., Jen B., Chiang P. (2024). A new AI-assisted data standard accelerates interoperability in biomedical research. medRxiv.

[54] Tran M., Balasooriya C., Jonnagaddala J., Leung G.K., Mahboobani N., Ramani S., Rhee J., Schuwirth L., Najafzadeh-Tabrizi N.S., Semmler C. (2025). Situating governance and regulatory concerns for generative artificial intelligence and large language models in medical education. NPJ Digit. Med..

[55] Krishnan S., Matheny M., Fontaine E., Adams L., National Academy of Medicine (2025). An Artificial Intelligence Code of Conduct for Health and Medicine: Essential Guidance for Aligned Action.

[56] https://www.fda.gov/media/184830/download.

